# Biological reliability of a movement analysis assessment using a markerless motion capture system

**DOI:** 10.3389/fspor.2024.1417965

**Published:** 2024-08-27

**Authors:** Nicolas M. Philipp, Andrew C. Fry, Eric M. Mosier, Dimitrije Cabarkapa, Justin X. Nicoll, Stephanie A. Sontag

**Affiliations:** ^1^ Jayhawk Athletic Performance Laboratory – Wu Tsai Human Performance Alliance, University of Kansas, Lawrence, KS, United States; ^2^ Kinesiology Department, Washburn University, Topeka, KS, United States; ^3^ Department of Kinesiology, California State University-Northridge, Los Angeles, CA, United States; ^4^ Applied Health and Recreation, School of Kinesiology, Oklahoma State University, Stillwater, OK, United States

**Keywords:** biomechanics, motion capture, movement screen, human movement, reliability

## Abstract

**Introduction:**

Advances in motion capture technology include markerless systems to facilitate valid data collection. Recently, the technological reliability of this technology has been reported for human movement assessments. To further understand sources of potential error, biological reliability must also be determined. The aim of this study was to determine the day-to-day reliability for a three-dimensional markerless motion capture (MMC) system to quantify 4 movement analysis composite scores, and 81 kinematic variables.

**Methods:**

Twenty-two healthy men (*n* = 11; $\bar{X}\;\pm \;\mathrm{SD}$; age = 23.0 ± 2.6 years, height = 180.4.8 cm, weight = 80.4 ± 7.3 kg) and women (*n* = 11; age = 20.8 ± 1.1 years, height = 172.2 ± 7.4 cm, weight = 68.0 ± 7.3 kg) participated in this study. All subjects performed 4 standardized test batteries consisting of 14 different movements on four separate days. A three-dimensional MMC system (DARI Motion, Lenexa, KS) using 8 cameras surrounding the testing area was used to quantify movement characteristics. 1 × 4 RMANOVAs were used to determine significant differences across days for the composite movement analysis scores, and RM-MANOVAs were used to determine test day differences for the kinematic data (*p* < 0.05). Intraclass correlation coefficients (ICCs) were reported for all variables to determine test reliability. To determine biological variability, mean absolute differences from previously reported technological variability data were subtracted from the total variability data from the present study.

**Results:**

No differences were observed for any composite score (i.e., athleticism, explosiveness, quality, readiness; or any of the 81 kinematic variables. Furthermore, 84 of 85 measured variables exhibited good to excellent ICCs (0.61–0.99). When compared to previously reported technological variability data, 62.3% of item variability was due to biological variability, with 66 of 85 variables exhibiting biological variability as the primary source of error (i.e., >50% total variability).

**Discussion:**

Combined, these findings effectively add to the body of literature suggesting sufficient reliability for MMC solutions in capturing kinematic features of human movement.

## 1 Introduction

With a substantial growth in the use and access to evolving technologies, sport science practitioners, as well as others working in the human movement and performance landscape now have the means to collect and communicate data in a more efficient manner than before. Within sports organizations, global positioning systems, quantifying athlete external practice and competition workloads, as well as force platforms and velocity-based training devices, allowing for the quantification of further kinetic and kinematic data are currently some of the most used means to quantify information on athletes. These data are often gathered concurrently as part of ongoing practices or training sessions, making for little interference with the athletes' already busy schedules. On the other hand, other means of gathering information such as motion capture screenings have historically presented with greater logistical challenges, requiring more dedicated time from both the athletes and practitioners. Therefore, technologies allowing for more efficient data collection procedures have been explored over recent years. While generally still considered as the gold-standard in the field of biomechanics, once completely laboratory-based, and requiring the often tedious and time-consuming attachment of reflective markers. Human motion-capture systems using markerless motion capture (MMC) solutions have gained increasing amounts of attention, especially in field-based settings such as sports environments, hospitals or physical therapy clinics (1). Further, when using marker-based motion capture technologies, the location of markers, as well as the movement of the skin with respect to the underlying tissues and bony landmarks, known as soft tissue artifact, may present challenges with regards to the acquisition of repeatable and valid movement data (2, 3). Likely however, the overarching benefit of MMC technologies lies in the efficient testing procedures, allowing for the capturing of more frequent data points, which may have positive implications for the health and performance of a broad range of populations (1, 4, 5). For instance, across a rehabilitation spectrum, more frequent data points may allow clinicians to better understand the patients' progress over time, enabling them to make more informed decisions. In line with this, Mauntel et al. (6) explored the reliability of a MMC system, used concurrently with a movement assessment software in scoring the landing error scoring system test (LESS), from which clinically important movement data may be derived. Authors suggested that the markerless system was able to reliably score the LESS test and provide results that were consistently accurate (5, 6).

These previously mentioned evolutions in sport science technologies, and the vast growth of data availability to enhance decision making in sport and health make it more important than before to critically evaluate the validity and reliability of the tools that are being used to take measurements (7). Previous groups have proposed three factors that contribute to a good performance test: (i) validity; (ii) reliability; and (iii) sensitivity (8). In line with the previous, reliability refers to the reproducibility of the values of a test (9), and can be influenced by variation in performance by the test subject (biological reliability), variation in the test methods, and variation in measurement of testing equipment (technological reliability) (10).

Still in its infant stages, some have suggested that this increase in test efficiency of MMC systems may come with a sacrifice in data reliability (11, 12). Others have suggested sufficient reliability of MMC systems (1, 13–17). Within a recent SWOT (i.e., Strengths, Weaknesses, Opportunities, Threats) analysis, authors suggested that a strength of low-cost MMC systems is the emerging agreement between them, when compared to marker-based systems (5). For instance, Sandau et al. suggested that a MMC system was able to reliably produce data within the sagittal and frontal plane of motion during a walking task, while data that was produced in the transverse plane showed lesser degrees of agreement, when compared to a traditional marker-based system (13). Similarly, Tanaka et al. proposed that while absolute measures in the hip joint angles obtained using MMC technology differed from marker-based technology, the MMC system presented usefulness in accurately classifying subjects' movement strategies adopted during a functional reach test (18). Further, Cabarkapa et al. highlighted the repeatability of motion-health screening score, derived from a MMC system, consisting of 8 cameras (16). More specifically, this study documented that algorithm-based motion health scores, which are often used by applied and clinical practitioners, showed moderate to excellent levels of agreement over six testing sessions (16). Hauenstein recently conducted a study, instigating the interrater (i.e., between-system agreement), and intrarater (i.e., agreement between first and second set within each system) agreement (17). Primary findings suggested that moderate [intraclass correlation coefficient (ICC), >0.50] to excellent (ICC, >0.90) reliability was displayed across all 39 kinematic variables analyzed in this study (17). Lastly, Philipp et al. recently emphasized the technological reliability of a MMC system, quantifying a range of elementary movement patterns, from which a vast number of kinematic metrics were derived (1). In this study, two identical MMC systems consisting of 8 total cameras, positioned in close proximity to each other were used to collect kinematic data on 29 different movements, from which 214 different metrics were derived (1). Primary results suggested that 95.7% of all metrics analyzed showed negligible or small between-device effect sizes, with 91.6% of all metrics showing moderate or better agreements, showing further promise with regards to the adoption of MMC systems (1).

While the previous study highlighted the technological reliability of a MMC system, further reliability research is warranted investigating the variation in performance induced by the test subject, often referred to as the biological variability or reliability. Therefore, the aim of the present study was to add further context to the reliability construct of MMC systems, by studying the day-to-day variability in kinematic metrics derived from a battery of different elementary movements, similar to previous research reports (1). Understanding the biological variability in addition to the technological variability will ultimately aid researchers in creating a more complete picture of the overall reliability of MMC technologies in capturing human movement data. Authors believe this to be the first of its kind exploration to understand human variability in motion testing through markerless solutions. Most often, variability is attributed to technology, however, this allows us to further examine the human element. Finally, it is probable that the complete division between technological and human variability may still be unclear, however, our data should help explain some of the tendencies regarding how much variability is possibly attributed to human variance.

Researchers hypothesized that metrics from respective movements would display sufficient amounts of repeatability and low degrees of variability between the four timepoints studied in this investigation.

## 2 Materials and methods

### 2.1 Experimental design

To determine the test-retest reliability of a MMC system across four visits, spanning over four weeks, a three-dimensional (3-D) video MMC system was used to compare kinematic features as well as movement analysis scores derived from a movement screen consisting of 14 different movement tasks. An experimental within subjects' design was used to compare biomechanical variables and scores over the four assessment timepoints. More specifically, comparisons were made on the same individual across all four time points.

### 2.2 Subjects

Eleven healthy, recreationally active women ($\bar{X}\pm \;\mathrm{SD}$; age = 20.8 ± 1.1 years., height = 172.2 ± 7.4 cm, weight = 68.0 ± 7.2 kg) and eleven men (age = 23.0 ± 2.6 years., height = 180.3 ± 4.8 cm, weight = 80.4 ± 7.3 kg) volunteered to participate in this investigation. All subjects were physically active a minimum of one hour for three days a week for at least the preceding three months. None of the participants reported a history of current or prior neuromuscular diseases or musculoskeletal injuries specific to the ankle, knee, or hip joints. Subjects demonstrated functional range of motion in hip, knee, ankle, and shoulder joints without limiting mechanical motion and performance during a vertical jump and running. This study was approved by the University's institutional review board for human subjects' research. Each subject read and signed an informed consent form and completed a health history questionnaire prior to participating.

### 2.3 Procedures

Procedures for this investigation were adapted from earlier research out of the same laboratory (1, 16). Each subject visited the laboratory for four visits. During the first visit, each subject signed an informed consent document, then performed the movement screen. During all four visits, subjects completed a 10-min standardized warm-up protocol prior to performing the movement screen. Each subject completed one session per week at the same time of day. The laboratory temperature (24–28°C) and humidity (38%–42%) remained in a consistent range for all test sessions. All subjects performed a total of 14 different movements, from which a total of 81 kinematic variables were extracted. Unilateral squats and forward lunges were performed on each leg respectively. Similarly, vertical jumps were performed bilaterally, as well as on each leg respectively, with jump height calculated as the difference between the estimated center of mass during the highest recorded frame of the jump and standing height. These variables (with the number of variables in parentheses) included range of motion in degrees for both the right and left shoulders (12), hips (20), knees (16), ankles (16), torso rotation, flexion and extension (3), and knee valgus (4). Also distances for lunge stride length (2) and center of mass displacement were measured (8). Additionally, four movement analysis scores were derived, which are calculated based on different kinematic features, using arbitrary calculations. Table 1, which is adapted from earlier research (1) shows a breakdown and explanation of the 19 different movement tasks. Data was quantified using a 3-D MMC system (DARI Motion, Lenexa, KS, USA) composed of eight high-definition cameras (Blackfly/FLIR GigE) recording at 60 fps. The cameras were attached to a metal frame surrounding the testing area, equidistant to each other. Further, the testing area consisted of a contrasting green floor, and no other persons or objects were allowed in the testing field during data collection. The hull technology model records and subtracts the visual signal minus the background, which is used to generate a pixelated person in order to obtain biomechanical parameters of interest. Following manufacturer guidelines, the system was calibrated prior to testing. Specific movement tasks were explained and demonstrated by the principal investigator of the study. Following this demonstration, the member of the research team running the motion capture system provided the subject with the following command: “three, two, one, go”. Following the “go” command, the subject completed the movement task which was being recorded by the 3-D MMC system. After the completion of the respective movement task, the command “done” was provided to the subject, to indicate the end of the movement. In line with earlier research, 3-D MMC joint coordinate systems were defined during movement tracking and calculations for all joints of interest follow the methods prescribed by the International Society of Biomechanics (19–21).

**Table 1:** List and description of all 14 tested movements (18 considering left and right for respective tests). Descriptions are listed how instructions were provided to the subjects.

Specific movement performed	Description of movement
Shoulder abduction	Start with arms at your sides with your palms facing forward. With arms straight, raise them out from your sides and over your head (abduct), keeping palms forward throughout the entire movement
Shoulder horizontal abduction	Start with your arms out in front of you at shoulder height with your palms facing each other. Bring your arms away from each other and behind your body as far as possible, keeping them at shoulder height throughout
Shoulder internal/external rotation	Start with elbows and shoulders bent at 90 degrees and palms facing down. Rotate arms up and back as far as possible (externally), and then forward and down (internally). Keeping elbows in the same spot during the movement
Shoulder flexion/extension	Begin with arms by your side. In one fluid motion, bring hands forward and up above the head, then down and back behind the body, and then return to original position.
Trunk rotation	Start with elbows and shoulders bent at 90 degrees and palms facing down. In one fluid motion, rotate arms, torso, and head, first to the right, then to the left, and then return to starting position
Body weight squat	Begin with feet shoulder width apart and toes pointing forward. In one fluid motion, squat as low as possible, then return to the starting position
Overhead squat	Begin with feet shoulder width apart, toes pointing forward and the dowel rod held above the head, with hands positioned wider than shoulders. In one fluid motion, squat as low as possible, and return to the original position
Forward lunge right	Begin by striding out with right leg getting as far and deep as possible. Then return to the starting position in one fluid motion. During movement keep arms out for balance
Forward lunge left	Begin by striding out with left leg getting as far and deep as possible. Then return to the starting position in one fluid motion. During movement keep arms out for balance
Unilateral squat right	Transfer weight to the right leg, lifting the left foot off the ground and behind the body. In one fluid motion, squat as low as possible, keeping the left foot off the ground, and arms out for balance
Unilateral squat left	Transfer weight to the right leg, lifting the left foot off the ground and behind the body. In one fluid motion, squat as low as possible, keeping the left foot off the ground, and arms out for balance
Vertical jump	Begin by standing with feet shoulder width apart. Load and jump as high as possible. Do not step into the jump, but you may use an arm swing
Static vertical jump	Begin by standing with feet shoulder width apart. Lower into a squat position with arms repositioned to a natural jumping stance. Remain in this position for two seconds. On the signal “jump” immediately jump as high as possible from the squat position
Unilateral vertical jump right	Begin by standing on right leg with left foot off the ground behind the body. Load and jump as high as possible, using an arm swing, and landing on your right foot again
Unilateral vertical jump left	Begin by standing on left leg with left foot off the ground behind the body. Load and jump as high as possible, using an arm swing, and landing on your left foot again
5 hop right	Begin standing on the right leg with left foot off the ground behind the body. Jump on the right leg five times. Jump as high as possible, and as fast as possible, spending as little time on the ground between jumps as possible
5 hop left	Begin standing on the left leg with right foot off the ground behind the body. Jump on the left leg five times. Jump as high as possible, and as fast as possible, spending as little time on the ground between jumps as possible
Drop vertical jump	Begin standing on a 30-centimeter-high box. With either foot, step off the box landing on two feet. Immediately jump for maximal height, spending as little time as possible on the ground. An arm swing may be used

### 2.4 Statistical analyses

Descriptive statistics, means and standard deviations (*x¯* ± SD), were calculated for each dependent variable. The individual movement analysis scores (i.e., athleticism, explosiveness, quality and readiness) for each test day were compared with a 1 × 4 repeated measures ANOVA. A one-way repeated measures multivariate analysis of variance (MANOVA), also referred to as a doubly multivariate MANOVA, was used to determine whether there were any statistically significant differences over time (Test 1–4) for multiple dependent variables classified in each of the fourteen distinct categories [i.e., left shoulder, right shoulder, trunk rotation, overhead squat, left unilateral squat, right unilateral squat, left forward lunge, right forward lunge, vertical jump (VJ), drop VJ, static VJ, left unilateral VJ, right unilateral VJ, multi-hop]. If the omnibus MANOVA test was significant, a follow-up analysis of variance (ANOVA) with post-hoc Bonferroni adjustments for multiple comparisons would have been carried out, but none were statistically significant. Mauchly's test for sphericity was not significant for any comparison. Two-way mixed intraclass correlation coefficients (ICC; two-way mixed effects model, subjects - random, tests – fixed, absolute agreement) were used to determine day-to-day reliability for each dependent variable (22). Additionally, absolute agreement for each of the repeated measurements for each dependent variable were used to determine the contributions of biological variability to the previously reported technological variability (1). Statistical significance was set *a priori* to *p *< 0.05. All statistical analyses were completed with SPSS (Version 29.0.0.0; IBM Corp., Armonk, NY, USA).

## 3 Results

Table 2 displays summary statistics for intraclass correlation coefficients (ICC). For the movement analysis assessments, no significant differences were observed for the following scores; athleticism (*F* = 0.010; df = 3,84; *p* = .999), explosiveness (*F* = 0.013; df = 3,84; *p* = .998), quality (*F* = 0.319; df = 3,84; *p* = .812), and readiness (*F* = 0.296; df = 3,84; *p* = .828). Omnibus MANOVAs indicated no statistically significant differences for any dependent variables for left shoulder (Wilks' *λ* = 0.821; df = 18.0, 223.9; *p* = 0.578), right shoulder (Wilks' *λ* = 0.805; df = 18.0, 223.9; *p* = 0.473), trunk rotation (Wilks' *λ* = 0.886; df = 6.0, 166.0; *p* = 0.119), overhead squat (Wilks' *λ* = 0.760; df = 30.0, 220.8; *p* = 0.856), left unilateral squat (Wilks' *λ* = 0.899; df = 15.0, 221.2; *p* = 0.889), right unilateral squat (Wilks' *λ* = 0.928; df = 15.0, 221.2; *p* = 0.976), left forward lunge (Wilks' *λ* = 0.870; df = 15.0, 221.3; *p* = 0.718), right forward lunge (Wilks' *λ* = 0.946; df = 15.0, 218.5; *p* = 0.995), vertical jump (VJ) (Wilks' *λ* = 0.876; df = 21.0, 224.5; *p* = 0.967), drop VJ (Wilks' *λ* = 0.889; df = 21.0, 224.5; *p* = 0.984), static VJ (Wilks' *λ* = 0.866; df = 21.0, 224.5; *p* = 0.946), left unilateral VJ (Wilks' *λ* = 0.944; df = 15.0, 221.2; *p* = 0.994), right unilateral VJ (Wilks' *λ* = 0.917; df = 15.0, 221.2; *p* = 0.953), and multi-hop (Wilks' *λ* = 0.897; df = 24.0, 223.9; *p* = 0.998) variables. Reliability as determined by ICCs resulted in good – excellent values (0.60–0.74 = good, 0.75–1.00 = excellent) (23) for athleticism, explosiveness, quality and readiness movement analysis scores (see Table 3). For the 81 individual dependent variables, ICCs for all but one variable were either good (11 variables) or excellent (69 variables). Only maximum right ankle flexion during landing from a drop jump exhibited a lower ICC (0.49, fair). When the mean absolute differences for all between test comparisons for each dependent variable were compared with previously reported mean absolute differences between two identical motion capture systems [technical variability; (1)], it was possible to determine how much of the total variability reported in the present study was due to biological variability of the subjects being tested (see Table 4). Biological variability ranged from 0.0%–97.9% of the total variability. When all dependent variables are combined, most of the variability is attributed to biological factors (technical variability = 33.1%, biological variability = 66.9%). In general, 37 dependent variables exhibited contributions from biological variability ≥80%, 28 dependent variables with contributions from biological variability ranging from >50%–79%, while only 16 dependent variables exhibited contributions that were primarily due to technical variability (i.e., technical variability > biological variability).

**Table 2:** Summary statistics for intraclass correlation coefficients (ICC).

ICC	Metric count (*n* = 85)	Total metrics	Metric count (% of total)	Total (%)
≥0.90	29	–	34.1%	–
≥0.80–0.89	41	70	48.2%	82.3%
≥0.70–0.79	8	78	9.4%	91.7%
≥0.60–0.69	6	84	7.0%	98.7%
≥0.50–0.59	0	84	0%	98.7%
≥0.40–0.49	1	85	1.2%	100%
≥0.30–0.39	0	–	–	100%
≥0.20–0.29	0	–	–	100%
<0.20	0	–	–	–

**Table 3:** Mean (±SD) values for segmental angles and ranges of motion for four different test sessions performed on separate days.

Anatomical motion or analysis score	ICC	Day 1	Day 2	Day 3	Day 4
Movement Analysis Scores					
Athleticism (*F* = 0.010; df = 3,84; *p* = .999)	0.92^a^	1,611.1 ± 307.8	1,611.8 ± 271.4	1,600.2 ± 270.9	1,601.5 ± 270.5
Explosiveness (*F* = 0.013; df = 3,84; *p* = .998)	0.93^a^	836.5 ± 216.9	836.4 ± 196.5	825.7 ± 197.9	833.5 ± 207.6
Quality (*F* = 0.319; df = 3,84; *p* = .812)	0.83^a^	907.3 ± 114.9	900.8 ± 106.8	885.6 ± 117.9	879.8 ± 100.5
Readiness (*F* = 0.296; df = 3,84; *p* = .828)	0.89^a^	18.7 ± 3.9	18.8 ± 3.6	18.6 ± 3.6	18.7 ± 3.4
Shoulder, left (Wilks’ Lambda = .821; df = 18.0,223.9; *p* = .578)					
Shoulder abduction mobility, maximum left (°)	0.87^a^	178.1 ± 11.0	179.1 ± 8.0	181.3 ± 10.1	180.9 ± 9.0
Shoulder horizontal abduction mobility, maximum left (°)	0.85^a^	88.3 ± 8.2	87.7 ± 8.8	83.0 ± 16.9	86.6 ± 16.5
Shoulder external rotation, maximum left (°)	0.85^a^	−88.7 ± 11.6	−92.5 ± 22.7	−88.2 ± 10.5	−89.1 ± 10.9
Shoulder internal rotation, maximum left (°)	0.80^a^	62.7 ± 13.5	75.5 ± 27.7	71.4 ± 13.5	72.3 ± 13.6
Shoulder flexion, maximum left (°)	0.82^a^	172.1 ± 15.9	170.2 ± 13.8	172.8 ± 11.5	170.1 ± 13.0
Shoulder extension, maximum left (°)	0.84^a^	−33.9 ± 12.3	−35.4 ± 12.8	−34.5 ± 13.9	−35.1 ± 12.0
Shoulder, right (Wilks’ Lambda = .805; df = 18.0,223.9; *p* = .473)					
Shoulder abduction mobility, maximum right (°)	0.66^a^	173.8 ± 8.0	177.7 ± 6.2	178.5 ± 9.0	179.9 ± 8.8
Shoulder horizontal abduction mobility, maximum right (°)	0.85^a^	84.0 ± 10.3	85.4 ± 10.0	80.1 ± 14.7	85.9 ± 17.3
Shoulder external rotation, maximum right (°)	0.90^a^	−92.7 ± 12.8	−95.0 ± 10.6	−93.3 ± 11.6	−94.0 ± 11.9
Shoulder internal rotation, maximum right (°)	0.85^a^	61.6 ± 12.8	72.9 ± 25.9	71.1 ± 11.3	70.8 ± 13.0
Shoulder flexion, maximum right (°)	0.85^a^	171.7 ± 17.7	169.8 ± 13.4	170.9 ± 11.6	169.5 ± 13.9
Shoulder extension, maximum right (°)	0.85^a^	−34.7 ± 12.9	−36.8 ± 10.6	−35.7 ± 14.5	−35.4 ± 12.7
Trunk rotation (Wilks’ Lambda = .886; df = 6.0,166.0; *p* = .119)					
Maximum left (°)	0.80^a^	80.3 ± 22.0	76.0 ± 17.4	77.0 ± 21.5	71.3 ± 16.0
Maximum right (°)	0.87^a^	79.5 ± 17.8	76.2 ± 14.6	73.5 ± 18.0	65.0 ± 16.0
Overhead squat (Wilks’ Lambda = .760; df = 30.0,220.8; *p* = .856)					
Overhead squat COM depth (cm)	0.97^a^	23.0 ± 3.7	23.9 ± 8.3	22.5 ± 3.5	22.0 ± 3.6
Overhead squat hip flexion, maximum left (°)	0.89^a^	128.5 ± 9.0	125.6 ± 10.3	126.7 ± 11.9	123.0 ± 10.3
Overhead squat hip flexion, maximum right (°)	0.89^a^	126.5 ± 9.1	125.5 ± 9.3	126.3 ± 11.2	123.4 ± 8.7
Overhead squat knee flexion, maximum left (°)	0.95^a^	133.6 ± 12.6	132.5 ± 15.1	131.8 ± 11.3	131.9 ± 11.9
Overhead squat knee flexion, maximum right (°)	0.96^a^	134.4 ± 13.7	133.5 ± 15.0	132.5 ± 10.4	132.3 ± 11.6
Overhead squat ankle flexion, maximum left (°)	0.84^a^	45.6 ± 7.0	44.8 ± 9.0	46.5 ± 8.0	47.3 ± 7.0
Overhead squat ankle flexion, maximum right (°)	0.81^a^	48.5 ± 6.8	47.7 ± 8.2	48.4 ± 8.1	49.8 ± 8.0
Overhead squat, trunk flexion (°)	0.87^a^	31.9 ± 11.1	29.6 ± 7.7	28.4 ± 11.3	31.4 ± 11.7
Overhead squat hip abduction, left (°)	0.91^a^	17.2 ± 7.0	18.0 ± 7.7	17.8 ± 9.7	17.1 ± 8.1
Overhead squat hip abduction, right (°)	0.92^a^	20.1 ± 7.8	20.1 ± 6.6	19.8 ± 8.5	17.1 ± 6.8
Unilateral squat, left (Wilks’ Lambda = .899; df = 15.0,221.2; *p* = .889)					
Unilateral squat COM depth, left (cm)	0.85^a^	14.9 ± 4.6	14.7 ± 2.6	14.2 ± 3.1	14.4 ± 2.6
Unilateral squat hip flexion, maximum left (°)	0.88^a^	106.7 ± 18.6	106.1 ± 13.0	105.4 ± 18.6	102.3 ± 16.8
Unilateral squat knee flexion, maximum left (°)	0.84^a^	101.9 ± 24.8	104.3 ± 7.9	104.7 ± 11.7	105.3 ± 10.0
Unilateral squat ankle flexion, maximum left (°)	0.85^a^	52.3 ± 5.6	51.6 ± 6.7	53.1 ± 6.2	53.6 ± 5.8
Unilateral squat dynamic valgus, left (°)	0.61^a^	5.4 ± 1.8	5.5 ± 1.8	8.7 ± 13.8	5.0 ± 3.2
Unilateral squat, right (Wilks’ Lambda = .928; df = 15.0,221.2; *p* = .976)					
Unilateral squat COM depth, right (cm)	0.89^a^	15.0 ± 3.4	15.0 ± 2.7	14.4 ± 3.4	14.0 ± 2.9
Unilateral squat hip flexion, maximum right (°)	0.84^a^	108.0 ± 14.7	108.3 ± 12.8	105.5 ± 16.0	104.7 ± 16.5
Unilateral squat knee flexion, maximum right (°)	0.88^a^	108.7 ± 12.4	107.3 ± 12.6	104.7 ± 13.2	105.0 ± 8.8
Unilateral squat ankle flexion, maximum right (°)	0.84^a^	49.1 ± 8.6	48.5 ± 8.6	49.3 ± 7.1	50.8 ± 7.8
Unilateral squat dynamic valgus, right (°)	0.75^a^	4.2 ± 2.8	5.3 ± 2.9	4.7 ± 2.9	5.6 ± 3.6
Forward lunge, left (Wilks’ Lambda = .870; df = 15.0,221.3; *p* = .718)					
Forward lunge stride length, left (cm)	0.82^a^	38.6 ± 5.3	37.1 ± 8.3	39.2 ± 5.9	39.2 ± 5.3
Forward lunge trail hip extension, left (°)	0.91^a^	−31.2 ± 16.4	−29.8 ± 13.2	−27.4 ± 13.2	−31.5 ± 14.5
Forward lunge hip flexion, maximum left (°)	0.71^a^	106.2 ± 13.4	102.2 ± 22.0	106.3 ± 16.8	106.7 ± 9.8
Forward lunge knee flexion, maximum left (°)	0.70^a^	122.3 ± 9.1	120.2 ± 12.5`	119.3 ± 5.1	120.1 ± 6.5
Forward lunge ankle flexion, maximum left (°)	0.62^a^	25.1 ± 9.9	19.0 ± 13.0	20.8 ± 10.8	22.8 ± 9.0
Forward lunge, right (Wilks’ Lambda = .946; df = 15.0,218.5; *p* = .995)					
Forward lunge stride length, right (cm)	0.96^a^	38.6 ± 6.1	38.3 ± 4.8	39.1 ± 5.9	38.8 ± 5.4
Forward lunge trail hip extension, right (°)	0.90^a^	−29.6 ± 17.6	−31.7 ± 15.5	−31.7 ± 12.8	−32.9 ± 15.0
Forward lunge hip flexion, maximum right (°)	0.88^a^	105.3 ± 12.1	104.8 ± 9.5	103.0 ± 15.0	105.6 ± 10.6
Forward lunge knee flexion, maximum right (°)	0.69^a^	119.1 ± 8.7	121.0 ± 7.3	119.0 ± 6.7	119.4 ± 6.4
Forward lunge ankle flexion, maximum right (°)	0.65^a^	23.1 ± 12.9	25.5 ± 12.7	23.3 ± 12.7	24.4 ± 10.6
Vertical jump (Wilks’ Lambda = .876; df = 21.0,224.5; *p* = .967)					
Vertical jump center of mass height (cm)	0.95^a^	19.3 ± 5.2	19.2 ± 5.9	18.7 ± 4.7	19.5 ± 5.7
Vertical jump downward phase hip flexion, maximum left (°)	0.92^a^	111.5 ± 14.9	116.5 ± 18.8	116.4 ± 17.9	115.0 ± 18.1
Vertical jump downward phase hip flexion, maximum right (°)	0.91^a^	111.3 ± 15.5	116.1 ± 19.3	116.5 ± 16.9	114.7 ± 17.6
Vertical jump downward phase knee flexion, maximum left (°)	0.92^a^	111.1 ± 24.5	118.2 ± 13.9	117.0 ± 13.6	118.5 ± 13.1
Vertical jump downward phase knee flexion, maximum right (°)	0.92^a^	113.0 ± 12.6	115.2 ± 14.4	114.7 ± 13.9	116.3 ± 13.2
Vertical jump downward phase ankle flexion, maximum left (°)	0.83^a^	42.5 ± 7.4	39.2 ± 9.0	39.0 ± 7.6	41.5 ± 8.3
Vertical jump downward phase ankle flexion, maximum right (°)	0.85^a^	40.6 ± 8.7	36.3 ± 11.9	37.9 ± 7.4	38.8 ± 10.1
Drop vertical jump (Wilks’ Lambda = .889; df = 21.0,224.5; *p* = .984)					
Drop jump height (cm)	0.99^a^	20.7 ± 5.2	20.4 ± 4.8	20.3 ± 4.5	20.1 ± 4.8
Drop jump landing hip flexion, left (°)	0.93^a^	87.7 ± 39.1	97.1 ± 36.4	104.9 ± 29.3	104.8 ± 34.5
Drop jump landing hip flexion, right (°)	0.93^a^	89.4 ± 38.0	94.5 ± 35.4	104.9 ± 29.3	103.9 ± 34.9
Drop jump landing knee flexion, left (°)	0.87^a^	106.0 ± 27.5	108.8 ± 18.8	113.8 ± 15.7	112.6 ± 26.6
Drop jump landing knee flexion, right (°)	0.83^a^	102.3 ± 34.2	106.5 ± 22.3	112.4 ± 13.3	111.5 ± 29.7
Drop jump landing ankle flexion, left (°)	0.84^a^	22.3 ± 38.5	21.0 ± 35.9	33.7 ± 24.6	36.1 ± 22.1
Drop jump landing ankle flexion, right (°)	0.49	25.3 ± 35.6	18.5 ± 35.1	29.5 ± 31.1	33.9 ± 22.4
Static vertical jump (concentric only) (Wilks’ Lambda = .866; df = 21.0,224.5; *p* = .946)					
Static VJ center of mass height (cm)	0.99^a^	18.8 ± 5.1	17.9 ± 4.5	17.9 ± 4.3	17.7 ± 4.4
Static VJ hip flexion, maximum left (°)	0.87^a^	113.5 ± 11.2	117.7 ± 9.8	114.2 ± 25.5	110.9 ± 23.3
Static VJ hip flexion, maximum right (°)	0.90^a^	114.5 ± 11.9	116.7 ± 10.7	118.7 ± 12.5	115.7 ± 11.6
Static VJ knee flexion, maximum left (°)	0.87^a^	114.8 ± 11.4	116.8 ± 12.6	117.4 ± 9.1	118.0 ± 11.3
Static VJ knee flexion, maximum right (°)	0.90^a^	113.7 ± 12.3	115.2 ± 13.8	115.3 ± 10.0	116.5 ± 11.8
Static VJ ankle flexion, maximum left (°)	0.70^a^	38.1 ± 7.0	34.8 ± 7.1	34.2 ± 7.1	37.0 ± 8.1
Static VJ ankle flexion, maximum right (°)	0.76^a^	36.9 ± 7.1	33.8 ± 9.5	33.2 ± 7.2	35.6 ± 8.0
Unilateral vertical jump, left (Wilks’ Lambda = .944; df = 15.0,221.2; *p* = .994)					
Unilateral VJ center of mass height, left (cm)	0.91^a^	13.3 ± 3.9	13.7 ± 3.4	13.3 ± 4.4	13.4 ± 4.6
Unilateral VJ hip flexion, maximum left (°)	0.93^a^	87.0 ± 19.7	84.0 ± 19.7	85.8 ± 21.4	84.8 ± 20.0
Unilateral VJ knee flexion, maximum left (°)	0.90^a^	85.2 ± 10.7	84.9 ± 10.0	87.3 ± 12.3	86.6 ± 11.1
Unilateral VJ ankle flexion, maximum left (°)	0.84^a^	44.7 ± 9.6	44.1 ± 9.1	44.0 ± 9.6	45.1 ± 9.4
Unilateral VJ dynamic valgus, left (°)	0.88^a^	0.6 ± 4.1	1.5 ± 4.2	1.4 ± 3.4	1.4 ± 3.8
Unilateral vertical jump, right (Wilks’ Lambda = .917; df = 15.0,221.2; *p* = .953)					
Unilateral VJ center of mass height, right (cm)	0.96^a^	12.8 ± 3.7	12.5 ± 3.4	12.5 ± 3.1	12.7 ± 3.5
Unilateral VJ hip flexion, maximum right (°)	0.93^a^	82.7 ± 19.8	79.7 ± 18.5	81.8 ± 19.7	79.7 ± 19.0
Unilateral VJ knee flexion, maximum right (°)	0.90^a^	85.9 ± 11.5	85.4 ± 13.9	86.7 ± 12.1	87.8 ± 11.6
Unilateral VJ ankle flexion, maximum right (°)	0.79^a^	44.1 ± 8.8	41.4 ± 9.2	44.0 ± 7.4	44.1 ± 10.1
Unilateral VJ dynamic valgus, right (°)	0.81^a^	0.6 ± 3.0	0.0 ± 3.6	1.4 ± 3.8	1.2 ± 3.6
Multi-Hop (Wilks’ Lambda = .897; df = 24.0,223.9; *p* = .998)					
Multi-hop downward phase hip flexion, maximum left (°)	0.90^a^	66.9 ± 22.3	68.8 ± 22.4	61.0 ± 26.2	64.5 ± 26.2
Multi-hop downward phase hip flexion, maximum right (°)	0.80^a^	69.0 ± 20.5	64.7 ± 21.1	53.6 ± 36.3	62.5 ± 27.5
Multi-hop downward phase knee flexion, maximum left (°)	0.71^a^	72.4 ± 18.9	74.0 ± 10.1	72.0 ± 14.6	71.5 ± 15.7
Multi-hop downward phase knee flexion, maximum right (°)	0.81^a^	77.2 ± 11.9	74.8 ± 11.9	71.9 ± 15.1	73.7 ± 15.9
Multi-hop downward phase ankle flexion, maximum left (°)	0.72^a^	36.5 ± 16.6	33.8 ± 12.1	32.7 ± 17.6	36.9 ± 16.4
Multi-hop downward phase ankle flexion, maximum right (°)	0.61^a^	41.1 ± 15.8	36.6 ± 15.5	35.4 ± 16.5	38.5 ± 12.5

No significant differences were observed for any variable as determined from MANOVA analyses (*p* > .05).

^a^ ICC = good – excellent (23).

**Table 4:** Mean absolute differences between test sessions for all kinematic variables, and the relative contributions of biological variability and technical variablility.

Anatomical Motion	Mean absolute difference		Contributions to variability (%)	
	Inter-Device^⧫^	Inter-Day	Technical^⧫^	Biological
Shoulder abduction				
Shoulder abduction mobility, maximum left value (°)	0.6	6.3	9.5%	90.5%
Shoulder abduction mobility, maximum right value (°)	0.6	7.5	8.0%	92.0%
Shoulder horizontal abduction				
Shoulder horizontal abduction mobility, maximum left value (°)	3.3	12.6	29.2%	73.8%
Shoulder horizontal abduction mobility, maximum right value (°)	2.4	14.7	16.3%	83.2%
Shoulder internal/external rotation				
Shoulder external rotation, maximum left value (°)	1.1	9.0	12.2%	87.8%
Shoulder external rotation, maximum right value (°)	0.3	13.0	2.3%	97.7%
Shoulder internal rotation, maximum left value (°)	0.1	12.0	0.8%	99.2%
Shoulder internal rotation, maximum right value (°)	1.0	11.8	8.5%	91.5%
Shoulder flexion/extension				
Shoulder flexion, maximum left value (°)	1.6	13.4	11.9%	88.1%
Shoulder flexion, maximum right value (°)	3.6	11.5	31.3%	68.7%
Shoulder extension, maximum left value (°)	0.9	11.9	7.6%	92.4%
Shoulder extension, maximum right value (°)	0.3	12.5	2.4%	97.6%
Trunk rotation				
Maximum left value (°)	1.3^a^	19.2	5.2%	94.8%
Maximum right value (°)	1.8^a^	15.6	11.5%	88.5%
Overhead squat				
Overhead squat COM depth value (cm)	1.9	1.9	100.0%	0.0%^b^
Overhead squat hip flexion, maximum left value (°)	5.9*	6.1	96.7%	3.3%^b^
Overhead squat hip flexion, maximum right value (°)	6.0	6.5	92.3%	7.7%^b^
Shoulder abduction				
Shoulder abduction mobility, maximum left value (°)	0.6	6.3	9.5%	90.5%
Shoulder abduction mobility, maximum right value (°)	0.6	7.5	8.0%	92.0%
Shoulder horizontal abduction				
Shoulder horizontal abduction mobility, maximum left value (°)	3.3	12.6	29.2%	73.8%
Shoulder horizontal abduction mobility, maximum right value (°)	2.4	14.7	16.3%	83.2%
Shoulder internal/external rotation				
Shoulder external rotation, maximum left value (°)	1.1	9.0	12.2%	87.8%
Shoulder external rotation, maximum right value (°)	0.3	13.0	2.3%	97.7%
Shoulder internal rotation, maximum left value (°)	0.1	12.0	0.8%	99.2%
Shoulder internal rotation, maximum right value (°)	1.0	11.8	8.5%	91.5%
Shoulder flexion/extension				
Shoulder flexion, maximum left value (°)	1.6	13.4	11.9%	88.1%
Shoulder flexion, maximum right value (°)	3.6	11.5	31.3%	68.7%
Shoulder extension, maximum left value (°)	0.9	11.9	7.6%	92.4%
Shoulder extension, maximum right value (°)	0.3	12.5	2.4%	97.6%
Trunk rotation				
Maximum left value (°)	1.3^a^	19.2	5.2%	94.8%
Maximum right value (°)	1.8^a^	15.6	11.5%	88.5%
Overhead squat				
Overhead squat COM depth value (cm)	1.9	1.9	100.0%	0.0%^b^
Overhead squat hip flexion, maximum left value (°)	5.9*	6.1	96.7%	3.3%^b^
Overhead squat hip flexion, maximum right value (°)	6.0	6.5	92.3%	7.7%^b^
Overhead squat knee flexion, maximum left value (°)	0.2	6.1	3.3%	96.7%
Overhead squat knee flexion, maximum right value (°)	1.2	5.1	23.5%	76.5%
Overhead squat ankle flexion, maximum left value (°)	2.5	5.7	43.9%	56.1%
Overhead squat ankle flexion, maximum right value (°)	2.9	6.0	48.3%	51.7%
Overhead squat, trunk flexion value (°)	0.5	6.3	7.9%	92.1%
Overhead squat hip abduction, left value (°)	0.2	4.2	4.8%	95.2%
Overhead squat hip abduction, right value (°)	0.1	3.9	2.6%	97.4%
Unilateral squat				
Unilateral squat COM depth, left value (cm)	0.3	2.0	15.0%	85.0%
Unilateral squat COM depth, right value (cm)	0.3	1.8	16.7%	83.3%
Unilateral squat hip flexion, maximum left value (°)	8.5	10.2	83.3%	16.7%^b^
Unilateral squat hip flexion, maximum right value (°)	8.5	10.2	83.3%	16.7%^b^
Unilateral squat knee flexion, maximum left value (°)	1.4	9.5	14.7%	85.3%
Unilateral squat knee flexion, maximum right value (°)	2.0	7.9	25.3%	74.7%
Unilateral squat ankle flexion, maximum left value (°)	1.0	4.0	25.0%	75.0%
Unilateral squat ankle flexion, maximum right value (°)	3.4	5.5	61.8%	38.2%^b^
Unilateral squat dynamic valgus, left value (°)	7.5	3.4	100.0%	0.0%^b^
Unilateral squat dynamic valgus, right value (°)	2.8	2.3	100.0%	0.0%^b^
Forward lunge				
Forward lunge stride length, left value (cm)	2.3	2.8	82.1%	17.9%^b^
Forward lunge stride length, right value (cm)	1.8	2.4	75.0%	25.0%^b^
Forward lunge trail hip extension, left value (°)	1.5	8.0	18.8%	81.2%
Forward lunge trail hip extension, right value (°)	1.8	9.4	19.1%	80.9%
Forward lunge hip flexion, maximum left value (°)	0.3	10.8	2.8%	97.2%
Forward lunge hip flexion, maximum right value (°)	1.1	8.7	12.6%	87.4%
Forward lunge knee flexion, maximum left value (°)	1.5	6.2	24.2%	75.8%
Forward lunge knee flexion, maximum right value (°)	0.1	4.8	2.1%	97.9%
Forward lunge ankle flexion, maximum left value (°)	0.4	8.9	4.5%	95.5%
Forward lunge ankle flexion, maximum right value (°)	1.1	9.5	11.6%	88.4%
Vertical jump				
Vertical jump center of mass height (cm)	0.4	1.8	22.2%	77.8%
Vertical jump downward phase hip flexion, maximum left value (°)	4.3	10.0	43.0%	57.0%
Vertical jump downward phase hip flexion, maximum right value (°)	6.1	9.8	62.2%	37.8%^b^
Vertical jump downward phase knee flexion, maximum left value (°)	1.8	9.8	18.4%	81.6%
Vertical jump downward phase knee flexion, maximum right value (°)	4.0	7.1	56.3%	43.7%^b^
Vertical jump downward phase ankle flexion, maximum left value (°)	2.1	6.2	33.9%	66.1%
Vertical jump downward phase ankle flexion, maximum right value (°)	4.5	6.9	65.2%	34.8%
Drop jump				
Drop jump landing ankle flexion, left value (°)	1.1	20.8	5.3%	94.7%
Drop jump landing ankle flexion, right value (°)	2.9	28.5	10.2%	89.8%
Drop jump landing knee flexion, left value (°)	2.7	13.2	20.5%	79.5%
Drop jump landing knee flexion, right value (°)	2.5	16.1	15.5%	84.5%
Drop jump landing hip flexion, left value (°)	3.7	16.3	22.7%	77.3%
Drop jump landing hip flexion, right value (°)	7.0	16.9	41.4%	58.6%
Static vertical jump (concentric only)				
Static VJ hip flexion, maximum left value (°)	4.3	11.4	37.7%	62.3%
Static VJ hip flexion, maximum right value (°)	6.1	7.4	82.4%	17.6%^b^
Static VJ knee flexion, maximum left value (°)	1.8	7.9	22.8%	77.2%
Static VJ knee flexion, maximum right value (°)	4.0	7.6	52.6%	47.4%^b^
Static VJ ankle flexion, maximum left value (°)	2.1	6.4	32.8%	67.2%
Static VJ ankle flexion, maximum right value (°)	4.5	6.6	68.2%	31.8%^b^
Unilateral vertical jump				
Unilateral VJ center of mass height, left value (cm)	0.1	2.0	5.0%	95.0%
Unilateral VJ center of mass height, right value (cm)	0.1	1.4	7.1%	92.9%
Unilateral VJ hip flexion, maximum left value (°)	4.4	10.4	42.3%	57.7%
Unilateral VJ hip flexion, maximum right value (°)	5.5	10.3	53.4%	46.6%^b^
Unilateral VJ knee flexion, maximum left value (°)	1.8	6.6	27.3%	72.7%
Unilateral VJ knee flexion, maximum right value (°)	0.6	7.4	8.1%	91.9%
Unilateral VJ ankle flexion, maximum left value (°)	2.4	6.8	35.3%	64.7%
Unilateral VJ ankle flexion, maximum right value (°)	1.2	7.0	17.1%	82.9%
Unilateral VJ dynamic valgus, left value (°)	3.0	3.2	93.7%	6.3%^b^
Unilateral VJ dynamic valgus, right value (°)	3.8	3.0	100.0%	0.0%^b^
Multi-Hop				
Multi-hop downward phase hip flexion, maximum left value (°)	5.8	14.3	40.6%	59.4%
Multi-hop downward phase hip flexion, maximum right value (°)	3.4	17.8	19.1%	80.9%
Multi-hop downward phase knee flexion, maximum left value (°)	3.2	10.0	32.0%	68.0%
Multi-hop downward phase knee flexion, maximum right value (°)	3.4	8.9	38.2%	61.8%
Multi-hop downward phase ankle flexion, maximum left value (°)	1.6	12.1	13.2%	86.8%
Multi-hop downward phase ankle flexion, maximum right value (°)	1.4	12.8	10.9%	89.1%
		Mean	33.1%	66.9%

^⧫^ Technical variability data from Philipp et al. (1).

^a^ Combined from thoracic and lumbar rotation.

^b^ Biological variability < technical variability.

* Difference between devices (*p* < .05).

## 4 Discussion

The aim of the present study was to investigate the day-to-day reliability in kinematic metrics derived from a battery of different elementary movements. Findings suggested no significant differences with regards to the four composite movement analysis scores, presenting with ICC values ranging from 0.83 to 0.93. While movement analysis scores, based on arbitrary algorithms may be of interest to most practitioners, fellow researchers may be interested in the reliability data of further, more isolated kinematic measures extracted from the different movement tasks. MANOVA results suggested no statistical significance for any of the fourteen distinct categories [i.e., left shoulder, right shoulder, trunk rotation, overhead squat, left unilateral squat, right unilateral squat, left forward lunge, right forward lunge, vertical jump (VJ), drop VJ, static VJ, left unilateral VJ, right unilateral VJ, multi-hop], into which kinematic variables were grouped. 80 out of 81 of the kinematic variables of interest demonstrated good to excellent levels of absolute agreement, with 69 out of 81 variables displaying excellent agreement. Out of all metrics of interest, 91.7% displayed ICCs of 0.70 or higher, which has previously been suggested as a benchmark for minimally acceptable ICC values in the field of exercise and sports science (24, 25). These findings are encouraging with regard to the biological (i.e., day-to-day) reliability of kinematic measures derived from a MMC system. This is in agreement with a recent study using the same MMC system, highlighting that all kinematic variables from a similar movement screen displayed at least moderate inter-, and intrarater agreement, with most variables presenting with excellent reliability (ICCs, >0.90) (17). Interestingly, in our study, the only variable displaying less than moderate agreement was right ankle flexion derived from the landing phase of a drop jump (ICC = 0.49). This variability between test days was not observed for left ankle flexion in the same task (ICC = 0.84), nor was it observed for either ankle during the downward phase of the vertical jump (ICC = 0.83, 0.85), often referred to as the eccentric phase. While speculative, this finding may be attributed to the more complex nature of the drop jump, especially when testing recreationally trained individuals, who perform plyometric exercises less frequently. Further, comparisons with technological reliability suggest that for ankle flexion during the drop jump landing, most of the variability in the data is attributed to biological variability (94.7%, 89.9%), rather than technological variability (5.3%, 10.2%), which should be considered when interpreting these results.

In line with the previous suggestions, authors want to highlight data presented in Table 3, where contributions to variability were compared between technological variability and biological variability, using data from an earlier study out of our laboratory (1). In the process of establishing reliability, it is important to distinguish between technological variation (i.e., error associated with the technology) and biological variation (i.e., error introduced from human sources) (26). Recent reviews have even called for a greater distinction between these forms of variability in the field of health and sports performance (27, 28). Our data suggests that most variation was attributed to error from human sources, with 37 dependent variables exhibiting contributions from biological variability ≥80%, 28 dependent variables with contributions from biological variability ranging from >50%–79%, while only 16 dependent variables exhibited contributions that were primarily due to technical variability (i.e., technical variability > biological variability). It seems difficult to determine why some variables displayed greater biological variability, while other displayed greater technological variability. However, authors hypothesize that the recreationally trained nature of our sample may in part contribute to the fact that most variation was attributed to error from human sources. Therefore, similar methodologies may be applied in other subject groups such as trained athletes. Further, our findings are in agreement with recent literature studying the interrater (technological variability) and intrarater (biological variability) using the same MMC system used in our investigation (17). Hauenstein et al. suggested that in their investigation, generally, intrarater reliability was lower than interrater reliability for the same measurement of consideration, highlighting that this could be partly attributed to the familiarization or warm-up effect between the first and second set, performed during the same visit to the laboratory (17). Our study therefore effectively adds to the body of knowledge by studying intrarater reliability across more test occasions, not taking place within the same day.

While our findings effectively contribute to our current understanding of the reliability of MMC solutions in studying human movement, a few limitations of our work, as well as avenues of future research should be acknowledged. For instance, similar to suggestions by Hauenstein et al., the good reliability between days in our study may in part be attributed to the generally healthy nature of our subjects. In many cases, movement screenings such as the ones used in our investigation are implemented in pathological populations such as athletes returning from injury. Such populations may exhibit alternative movement characteristics (e.g., between-limb asymmetries) that were not observed in our subject pool. Therefore, future investigations should aim to study technological and biological reliability of MMC systems, in populations with abnormal movement characteristics (e.g., injured individuals).

In summary, our findings documented good between-day reliability for a number of kinematic variables extracted from a movement screen consisting of 14 different movements. Movement analysis scores based on proprietary algorithms displayed ICCs ranging from 0.83 to 0.93, while out of 81 other joint-, and movement-specific kinematic variables, 80 displayed good to excellent agreement, with only one variable presenting with less than moderate agreement. Looking at the overall sources of variability, our data suggests that most variation was attributed to error from human sources, with 37 dependent variables exhibiting contributions from biological variability ≥80%, 28 dependent variables with contributions from biological variability ranging from >50%–79%, while only 16 dependent variables exhibited contributions that were primarily due to technical variability (i.e., technical variability > biological variability). Combined, these findings effectively add to the body of literature suggesting sufficient reliability for MMC solutions in capturing kinematic features of human movement. Caution is advised when trying to generalize findings of this study to other technologies or populations. Regardless of limitations highlighted in our study, authors believe that findings may be of interest to practitioners working in the human movement landscape. More specifically, the extensive number of kinetic and kinematic variables studied provides readers with the opportunity to determine the reliability of variables they may deem important within their own settings.

## Data Availability

The raw data supporting the conclusions of this article will be made available by the authors, without undue reservation.
